# Genomic analysis of single nucleotide polymorphisms in malaria parasite drug targets

**DOI:** 10.1186/s13071-022-05422-4

**Published:** 2022-08-30

**Authors:** Jasmita Gill, Amit Sharma

**Affiliations:** 1 ICMR-National Institute of Malaria Research, Sector 8, Dwarka, 110077 New Delhi India; 2 International Centre for Genetic Engineering and Biotechnology, Aruna Asaf Ali Marg, New Delhi, 110067 India

**Keywords:** Aminoacyl-tRNA synthetases, SNPs, Lysyl, -prolyl and phenylalanyl-tRNA synthetases, Field isolates, MalariaGEN

## Abstract

Malaria is a life-threatening parasitic disease caused by members of the genus *Plasmodium*. The development and spread of drug-resistant strains of *Plasmodium* parasites represent a major challenge to malaria control and elimination programmes. Evaluating genetic polymorphism in a drug target improves our understanding of drug resistance and facilitates drug design. Approximately 450 and 19 whole-genome assemblies of *Plasmodium falciparum* and *Plasmodium vivax*, respectively, are currently available, and numerous sequence variations have been found due to the presence of single nucleotide polymorphism (SNP). In the study reported here, we analysed global SNPs in the malaria parasite aminoacyl-tRNA synthetases (aaRSs). Our analysis revealed 3182 unique SNPs in the 20 cytoplasmic *P. falciparum* aaRSs. Structural mapping of SNPs onto the three-dimensional inhibitor-bound complexes of the three advanced drug targets within aaRSs revealed a remarkably low mutation frequency in the crucial aminoacylation domains, low overall occurrence of mutations across samples and high conservation in drug/substrate binding regions. In contrast to aaRSs, dihydropteroate synthase (DHPS), also a malaria drug target, showed high occurrences of drug resistance-causing mutations. Our results show that it is pivotal to screen potent malaria drug targets against global SNP profiles to assess genetic variances to ensure success in designing drugs against validated targets and tackle drug resistance early on.

**Graphical Abstract:**

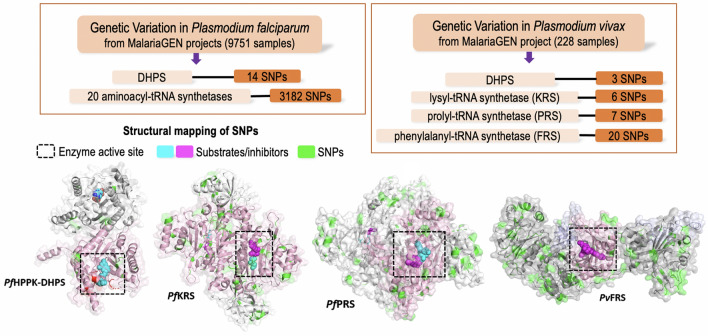

**Supplementary Information:**

The online version contains supplementary material available at 10.1186/s13071-022-05422-4.

## Introduction

Malaria is caused by *Plasmodium* parasites and is a life-threatening disease that remains an important public health concern in developing countries [1]. Eliminating malaria is complicated by the development and spread of resistance in the parasites *Plasmodium falciparum* and *Plasmodium vivax* to antimalarial drugs via the emergence of resistant strains [2]. The presence of parasites that are able to evade first-line antimalarial artemisinin-based combination therapies (ACTs) has been confirmed in malaria-endemic countries of South East Asia and Africa, posing a threat of disease resurgence [3]. To prevent health emergencies due to this common yet treatable disease, validation of newer drug targets and the design of novel antimalarial scaffolds are urgently needed [4, 5]. The aminoacyl-tRNA synthetase (aaRS) family of enzymes are universally distributed as they catalyse the linkage of cognate amino acid to transfer RNA (tRNA) that corresponds to the anti-codon triplet of the tRNA based on the genetic code [6]. During the aminoacylation reaction, ATP activates the amino acid by forming an aminoacyl-adenylate intermediate, following which this intermediate is ligated to the tRNA molecule and the final product is transported to the ribosome to carry out protein translation. Since aaRSs implement the genetic code for protein translation, their inhibition results in ribosome stalling during protein synthesis. The aaRS enzymes are validated and potent malaria drug targets [7–10]. Several three-dimensional (3D) structures of *Plasmodium* aaRSs are available in complex with substrates and/or inhibitors elucidating mechanistic details [11–18]. Thus, aaRSs provide a robust starting platform for the development of drugs against malaria.

Global field isolates of *Plasmodium* parasites from various locations worldwide are a valuable resource for understanding genetic variances in the parasite genome across populations. A single nucleotide polymorphism (SNP), which is a genomic variant at a single base position in the DNA, is the most common genetic variation that can alter the surface properties of a protein. The Malaria Genomic Epidemiology Network (MalariaGEN), a public data-sharing network with global study partners, integrates epidemiology with population genomics pertaining to malaria parasites [19]. MalariaGEN provides nucleotide-level SNP data for *P. falciparum* and *P. vivax* which have been obtained from clinical blood samples collected from malaria patients worldwide [19–23]. Within the framework of MalariaGEN, 9751 unique samples have been sequenced at multiple locations in over 50 countries in various projects dating from 2015 to 2020, resulting in the identification of approximately  8,051,696 SNPs in the genome of the *P. falciparum*, with the 3D7 strain as reference [20–22]. Additionally, 303,616 SNPs have been identified in the genome of *P. vivax,* with the Salvador 1 (Sal 1) strain as reference [23].

In this work, we have analysed global non-synonymous SNPs (referred to further as SNPs) in the 20 cytoplasmic *Plasmodium* aaRSs. Our analysis revealed 3182 unique SNPs in these 20 cytoplasmic *P. falciparum* aaRSs, with an average of 159 SNPs and 9–31% mutation frequency for individual aaRSs. Structural mapping of SNPs onto the inhibitor/substrate-bound 3D structures of three aaRSs, namely the *P. falciparum* lysyl (*Pf*KRS)-, prolyl (*Pf*PRS)- and *P. vivax* phenylalanyl (*Pv*FRS)-tRNA synthetases, showed a very low frequency of mutations in their catalytic domains and high conservation in the substrate/drug binding regions. In contrast, a similar analysis of another key malaria drug target, dihydropteroate synthase (DHPS), showed the presence of all drug resistance-causing mutations despite low overall mutation frequency.

Inherent variability in genomic sequences of drug and vaccine targets can be a shortcoming in the design and development of effective therapies as the targets will be naturally resistant (compromised) [24, 25]. Any amino acid change, particularly at the active site of a drug target, can induce alterations in the structural features which will alter its interaction with the drug and can lead to weak or no binding. Such polymorphisms can then lead to the rise of subgroups in a population which exhibit drug resistance. Based on the results of this study, the authors recommend investigating region- and population-specific polymorphisms in the ligand/substrate/inhibitor binding regions of drug targets to ensure success in therapeutic design against malaria.

## Methods

### SNP data

Data on SNPs were accessed from the following releases of MalariaGEN network (www.malariagen.net/data):

- The Pf3k Project (2016): pilot data release 5 (www.malariagen.net/data/pf3k-5). The non-synonymous (amino acid mutations) variants for each of the 20 *Pf*-aminoacyl-tRNA synthetases were noted from the data app available at https://www.malariagen.net/apps/pf3k/release_3/index.html by searching with Gene IDs (Additional file 1: Table S1). Details on a total of 2640 samples, including country and sites, are available at ftp://ngs.sanger.ac.uk/production/pf3k/release_5/ (Additional file 2: Table S2).
- *Plasmodium falciparum* Community Project—Catalogue of Genetic Variation v6.0 (www.malariagen.net/data/catalogue-genetic-variation-p-falciparum-v6.0 as described in MalariaGEN et al., Wellcome Open Research 2021642 and Catalogue of Genetic Variation v4.0 (www.malariagen.net/data/catalogue-genetic-variation-p-falciparum-v4.0). The non-synonymous (amino acid mutations) variants were noted from the data app available at https://www.malariagen.net/apps/pf/4.0/. A total of 7111 unique samples were available from the Pf4.0 and Pf6.0 projects. Thus, the total number of unique *P. falciparum* samples from which SNPs were gathered in this study are 9751 (2640 from the Pf3k project and 7111 from the Pf4.0 and Pf6.0 projects). For v6.0, Variant Call Format files (VCF) were downloaded using FTP site ftp://ngs.sanger.ac.uk/production/malaria/pfcommunityproject/Pf6/Pf_6_vcf/. VCF files were processed using BCFTools (www.samtools.github.io/bcftools/) on a MacOS (Apple Inc., Cupertino, CA, USA). Comma-separated value (.CSV) files were generated for each chromosome using BCFTools scripts available at ftp://ngs.sanger.ac.uk/production/malaria/pfcommunityproject/Pf6/Pf_6_vcf/About-Pf7_data.txt. The amino acid mutations that resulted from a gene mutation were taken from the .CSV file by extracting column “SNPEFF_AMINO_ACID_CHANGE”. The details of 7111 samples, including country and sites, are available at ftp://ngs.sanger.ac.uk/production/malaria/pfcommunityproject/Pf6/Pf_6_vcf/Pf_6samples.txt.
- MalariaGEN *Plasmodium* *vivax* Genome Variation project (2016). *Plasmodium vivax* Genome Variation May 2016 data release described in Pearson et al. [23] and https://www.malariagen.net/parasite/p-vivax-genome-variation [20–22]. The non-synonymous (amino acid mutation) variants for the DHPS domain of *Pv*HPPK-DHPS and three *P. vivax* aaRSs (*Pv*KRS, *Pv*PRS and *Pv*FRS) were noted from the data app available at https://www.malariagen.net/apps/pvgv/index.html by searching using Gene IDs (Additional file 1: Table S1). The details of 228 samples, including country and sites, are available at https://www.malariagen.net/parasite/p-vivax-genome-variation (Additional file 2: Table S2).

### Sequence and structural analysis

Three-dimensional structures were accessed from the Protein Data Bank (www.rcsb.org). Sequence analysis was done using Clustal Omega (www.ebi.ac.uk/Tools/msa/clustalo/). PDBsum (www.ebi.ac.uk/pdbsum/) was used for secondary structure analysis. All bar graphs and pie charts were made in Microsoft Excel (Microsoft Corp., Redmond, WA, USA). PLIP (www.plip-tool.biotec.tu-dresden.de/plip-web/plip/index) was used for analysing protein-ligand interactions. Structural observations were depicted using Pymol (www.pymol.org).

## Results and discussion

### SNP profiles of *P. falciparum* and *P. vivax* DHPS

**Fig. 1:**
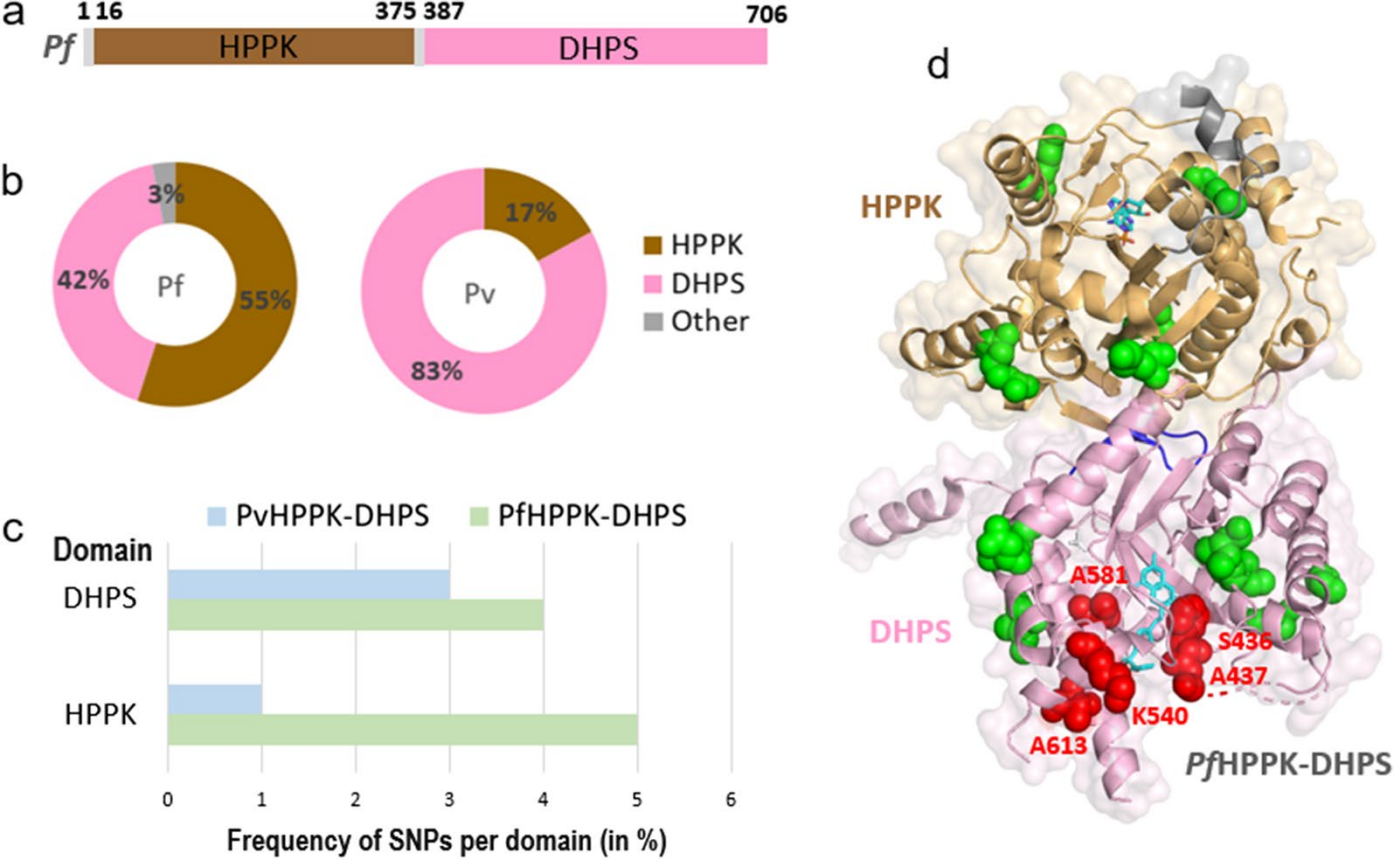
Single nucleotide polymorphism (SNP) profiles of *Plasmodium falciparum* HPPK-DHPS.** a** Domain diagram of *P. falciparum* HPPK-DHPS enzyme, with the HPPK and DHPS domains shown as brown and pink respectively. **b** Percentage distribution of amino acid mutations across the HPPK and DHPS domains in *Pf*HPPK-DHPS (left) and *Pv*HPPK-DHPS (right). **c** The percentage frequency of mutations within the HPPK and DHPS domains in *Pf*HPPK-DHPS and *Pv*HPPK-DHPS [(total number of amino acid mutations divided by total number of residues within the domain) × 100]. **d** Structural mapping of SNPs on the three-dimensional structure of *Pf*HPPK-DHPS (PDB ID: 6JWW), with the HPPK and DHPS domains shown as tan and pink ribbons and transparent surface. SNPs are shown as green spheres. The bound sulfathiazole (STZ) in DHPS domain and AMP in HPPK domain are shown as cyan sticks. The residues that undergo mutations in the DHPS domain are shown as red spheres. Abbreviations: *Pf*HPPK-DHPS,* Plasmodium falciparum* 6-hydroxy-methyl-7,8-dihydropterin pyrophosphokinase–dihydropteroate synthase; * Pv*HPPK-DHPS, *Plasmodium vivax* HPPK-DHPS; SNP, single nucleotide polymorphism

*Plasmodium* 6-hydroxy-methyl-7,8-dihydropterin pyrophosphokinase–dihydropteroate synthase (HPPK-DHPS) is a bifunctional fused enzyme that is essential for the biosynthesis of folate. Inhibition of this process results in depletion of dTTP, leading to decreased DNA synthesis in the parasites and ultimately in their death [26–28]. The DHPS domain of *Pf*HPPK-DHPS is inhibited by sulfadoxine (Sdx) and sulfa-class of compounds; however, the emergence of Sdx resistance is well-documented in field isolate studies worldwide [26–28]. Structures of *P*. *falciparum* and *P. vivax* HPPK-DHPS are available in complex with substrates/analogues (PDB ID: 6JWW and 5Z79) [27, 28]. *Pf*HPPK-DHPS is a dimer having HPPK and DHPS domains connected by a linker (Fig. 1a). Our analysis revealed 33 unique SNPs in *Pf*HPPK-DHPS, with an overall mutation frequency of 5% for a total of 706 residues (Fig. 1b, Additional file 3: Table S3). The *Pf*DHPS domain in itself has a lower frequency of 4% as it contains only 14 SNPs in its 320 residues (Fig. 1c). Further, *Pv*HPPK-DHPS contains an equivalent of 12 mutations as well as an additional five from the *P. vivax* project, of which two are unique. Of the five mutations from *P. vivax* project, four lie in the DHPS domain, with three of the latter known to be drug resistance-causing mutations. Similar low frequencies of mutations were seen in the *P. falciparum* and *P. vivax* DHPS domains (Fig. 1c). SNPs in the *Pf*DHPS domain are primarily hydrophobic/polar/charged residues mutating to hydrophobic/polar residues. No mutations were seen wherein a residue had mutated to a charged residue. A particular SNP in *Pf*DHPS domain was seen in at least one sample and in a maximum of 1892 samples (of the total 9751 unique samples from MalariaGEN projects Pf3k, Pf4.0 and Pf6.0). All five drug resistance-causing mutations in *P. falciparum* (S436A/F, A437G, K540E, A581G, and A613S/T) are prominently present in *Pf*DHPS domain in up to 1892 samples out of the 9751 MalariaGEN projects samples analysed. At least two drug resistance-causing mutations were found to coexist in 2552 samples out of 9751 (26% samples). Also, three mutations coexist in 97 samples, with the third mutation (E189Q) located in the HPPK domain. Structural mapping of SNPs on *Pf*HPPK-DHPS shows the presence of the drug resistance-causing mutations in the sulfa drug binding site (Fig. 1d), as reported earlier [29]. Moreover, the high occurrence of these mutations in samples confirms the considerable prevalence of Sdx resistance and underscores the importance of global SNP databases as keys to understanding ongoing resistance spread.

### SNP profiles of cytoplasmic *P. falciparum* aminoacyl-tRNA synthetases

**Fig. 2:**
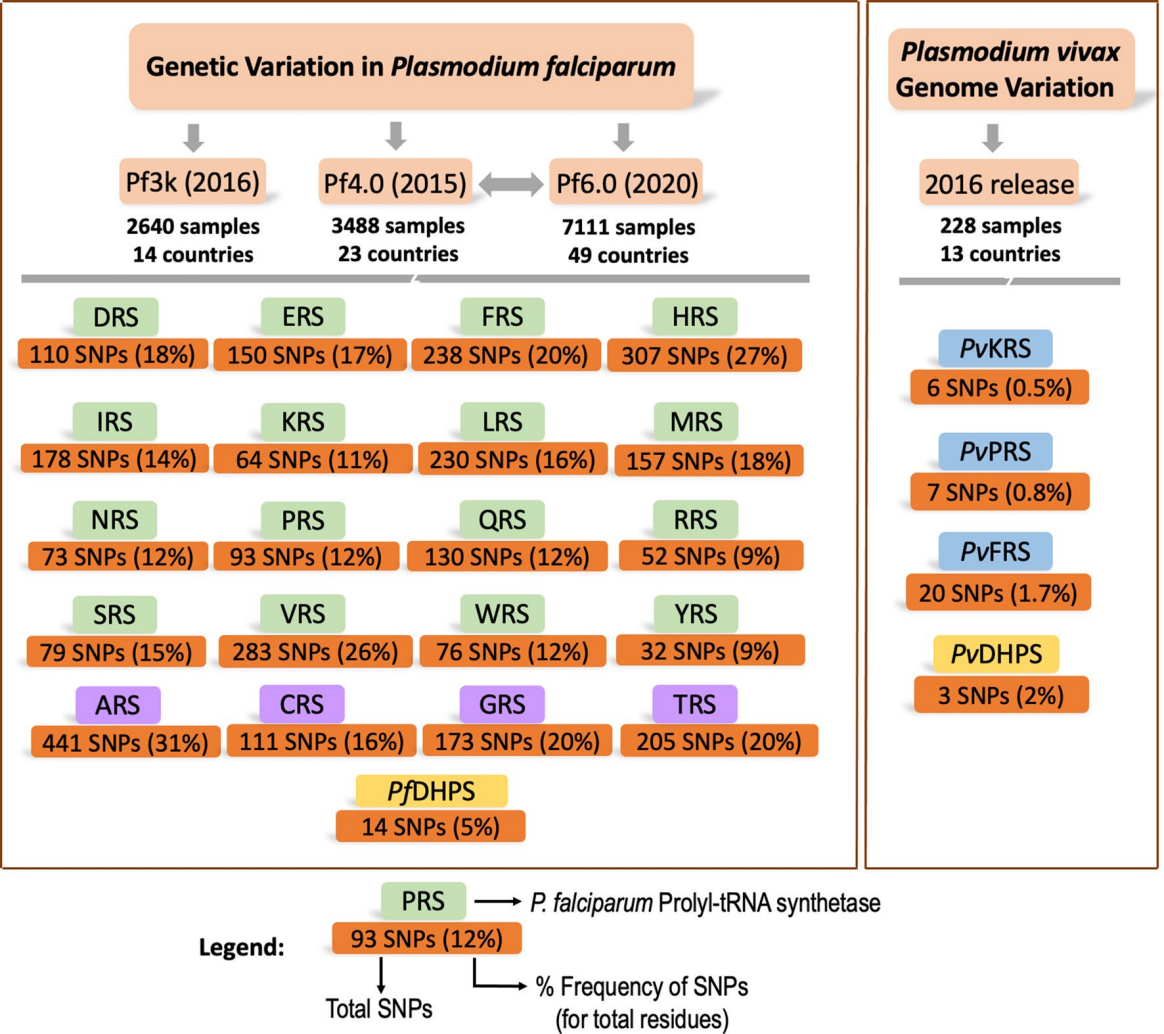
Data collection of SNPs from MalariaGEN. The *P. falciparum* and *P. vivax* sequencing projects of MalariaGEN are listed together with the number of samples collected and number of countries where the samples were collected at various sites (Additional file 2: Table S2). There was a total of 9751 unique *P. falciparum* samples (2640 from the Pf3k project and 7111 from the Pf4.0 and Pf6.0 projects). The 16 cytoplasmic *Pf*-aaRSs are shown in green boxes, with the first letter corresponding to the amino acid code (e.g.: prolyl-tRNA synthetase is PRS). The four dual localised *Pf*-aaRSs (ARS, GRS, TRS, CRS) are shown in mauve boxes. For the *P. vivax* project, three advanced cytoplasmic *Pv*-aaRSs (KRS, PRS and FRS) are shown in blue boxes. *Pf*DHPS and *Pv*DHPS domains are shown in a yellow box. The orange boxes below each enzyme show the total SNPs (amino acid mutations) and the percentage frequency of SNPs is given in parentheses [(total number of amino acid mutations divided by total number of residues in the enzyme) × 100]. Abbreviations: aaRS, aminoacyl-tRNA synthetase; DHPS, dihydropteroate synthase; MalariaGEN, Malaria Genomic Epidemiology Network; *Pf*, *Plasmodium falciparum*; *Pv*, *Plasmodium vivax*

**Fig. 3:**
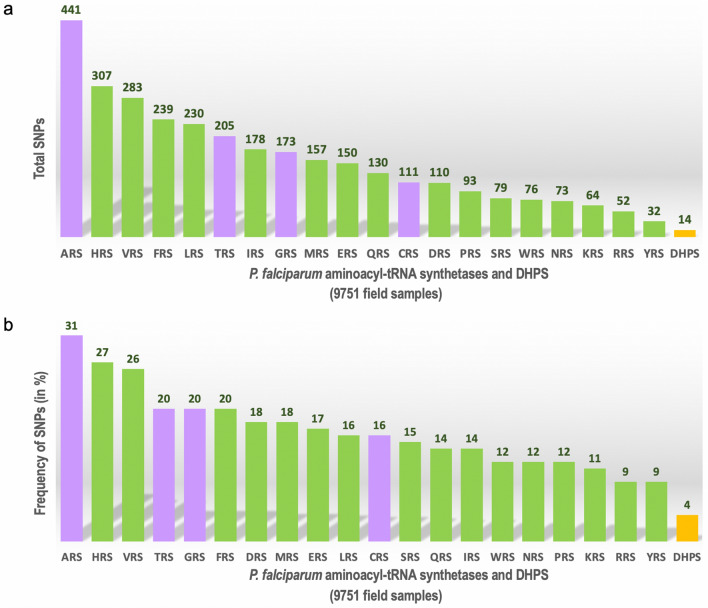
Graphical representation of SNPs in 20 *Pf-*aaRSs and *Pf*DHPS domain. **a** Total SNPs in 20 [16 cytoplasmic (green bars); 4 dual localised (mauve bars)] *Pf*-aaRSs and *Pf*DHPS domain (yellow bar) (Additional file 1: Table S1). There were a total of 9751 unique *P. falciparum* samples (2640 from the Pf3k project and 7111 from the Pf4.0 and Pf6.0 MalariaGEN projects). **b** Percentage frequency of amino acid mutations in 20 *Pf*-aaRSs and *Pf*DHPS domain [(total number of amino acid mutations divided by total number of residues in the enzyme) × 100]

*Plasmodium falciparum* has 36 aaRSs, of which 16 are cytoplasmic, 15 are apicoplastic, one is mitochondrial and four share localisations between the cytoplasm and the apicoplast [9, 10]. The 20 cytoplasmic *Plasmodium* aaRSs are potential drug targets for malaria, of which the 3—lysyl (*Pf*KRS)-, prolyl (*Pf*PRS)- and phenylalanyl (*Pv*FRS)-tRNA synthetases are highly advanced targets since several chemical, structural and activity-relationship datasets are available for these (Additional file 1: Table S1) [11–18]. Our analysis revealed a total of 3182 unique SNPs in the 20 *Pf*-aaRSs, with the highest frequency of mutations seen in the *Pf* ARS, HRS and VRS (Figs. 2, 3) and the lowest frequency of SNPs seen in the *Pf* WRS, NRS, PRS, KRS, RRS and YRS (Figs. 2, 3). Consequently, the average SNP frequency ranges from 9% to 31%, with an overall SNP frequency average of 5%. Analysis of the *P. vivax* genome sequences revealed 33 SNPs in *Pv*KRS, *Pv*PRS and *Pv*FRS (Fig. 2). 3D structures of cytoplasmic *Pf*KRS, *Pf*PRS and *Pv*FRS are available in complex with substrates and inhibitors [11–18]. Given that we have structures where *Pf*KRS is bound to inhibitor cladosporin and substrate L-Lys, *Pf*PRS is bound to inhibitor halofuginone and *Pv*FRS is bound to inhibitor bicyclic azetidine, these data will help in drug development against aaRSs. Structural mapping of SNPs can reveal their distribution and location on the protein surface. Interestingly, *Pf*KRS and *Pf*PRS have some of the lowest mutation frequencies among the *Pf*-aaRSs (Figs. 2, 3).

### SNP profiles of cytoplasmic *P. falciparum* and *P. vivax* lysyl-tRNA synthetase

**Fig. 4:**
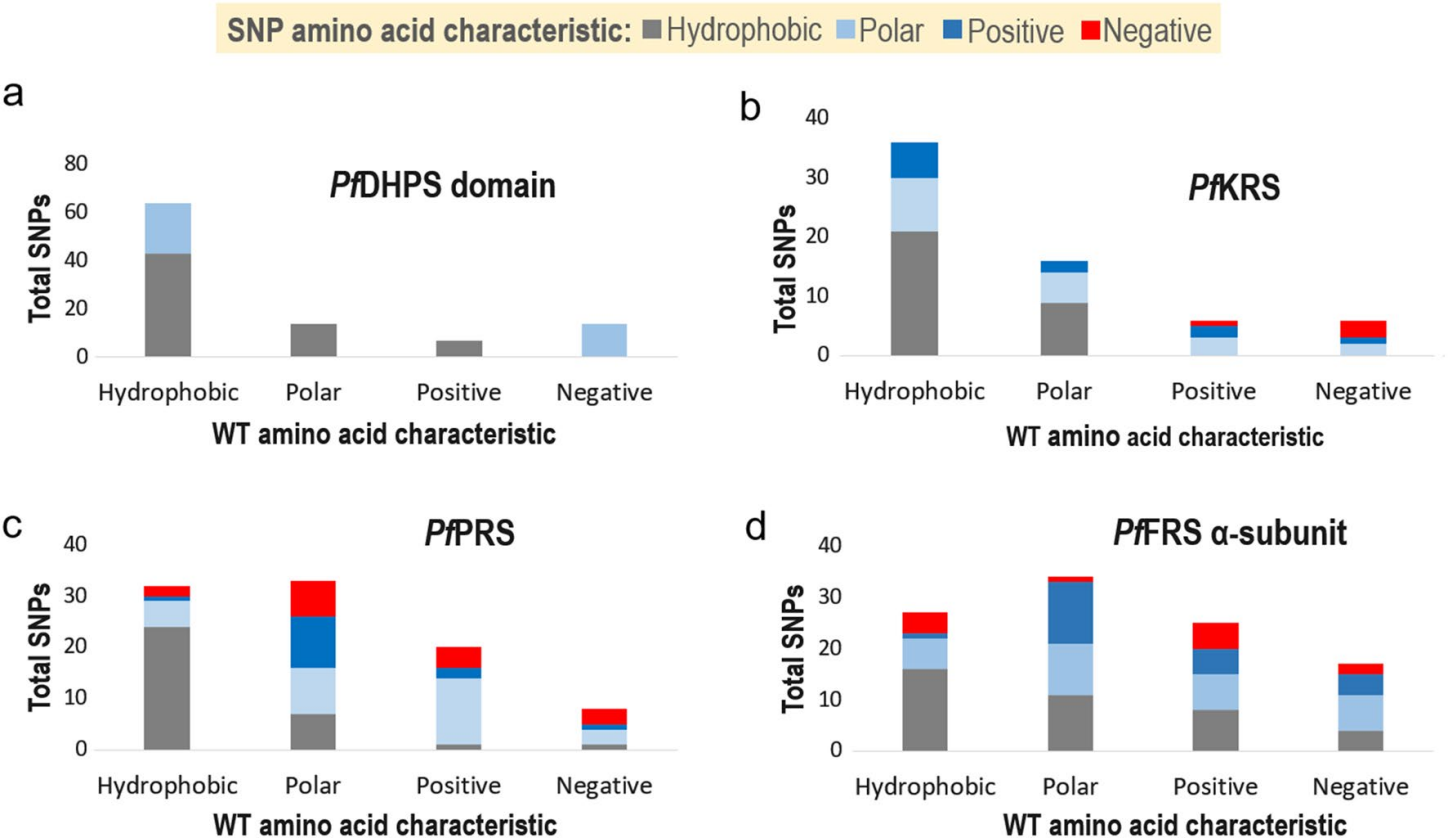
Graphical representation of types of SNPs in the *Pf*DHPS, *Pf*KRS, *Pf*PRS and *Pf*FRS α-subunit.** a**–**d** Distribution of SNPs based on their type in: **a** *Pf*DHPS domain of *Pf*HPPK-DHPS, **b** *Pf*KRS, **c** *Pf*PRS and **d** *Pf*FRS α-subunit. Residue classification is: hydrophobic (glycine, alanine, valine, leucine, isoleucine, proline, methionine, phenylalanine and tryptophan), polar (serine, threonine, cysteine, asparagine, tyrosine and glutamine), positive (histidine, arginine and lysine) and negative (aspartic acid and glutamic acid). The total number of mutations in which residues mutate to the hydrophobic state (grey bar), polar state (light-blue bar) or positive charge and negative charge (blue and red bars, respectively) is shown. Abbreviations: *Pf*DHPS, *P. falciparum* dihydropyeroate synthase; *Pf*KRS, *P. falciparum* lysyl-tRNA synthetase; *Pf*PRS, *P. falciparum* prolyl-tRNA synthetase; *Pf*FRS, *P. falciparum* phenylalanyl-tRNA synthetase

**Fig. 5:**
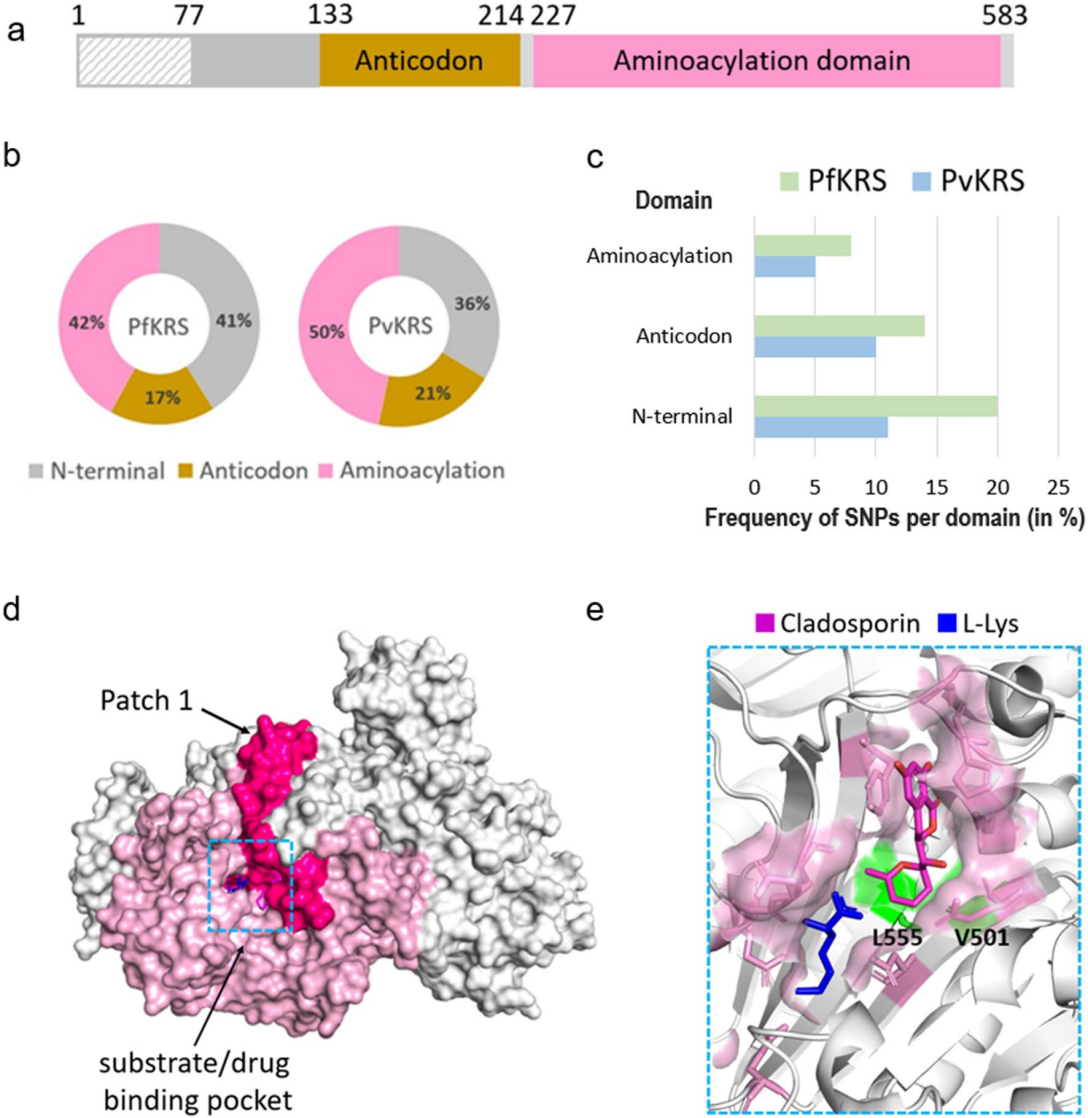
SNP profiles of cytoplasmic *Pf*KRS. **a** Domain diagram of *Plasmodium* KRS. Aminoacylation and anticodon domains are shown in pink and brown respectively; the N-terminal is shown in grey; and the disordered region in three-dimensional structures of *Pf*KRS is shown as grey patterning. **b** The percentage distribution of SNPs (amino acid mutations) across the domains in *Pf*KRS (left) and *Pv*KRS (right). **c** The percentage frequency of amino acid mutations per domain in *Pf*KRS and *Pv*KRS [(total number of amino acid mutations divided by total number of residues in the enzyme) × 100]. **d** Structure of the *Pf*KRS dimer surface (PDB ID: 4PG3). For simplicity, the aminoacylation domain of only one monomer is coloured light pink. The sequence patch 1 (amino acids 287–354), with no mutations, is colored dark pink. Substrate/inhibitor binding pocket is indicated with a blue box. **e** Close-up view of the primary binding pocket of *Pf*KRS with the bound inhibitor cladosporin (magenta stick) and substrate L-Lys (blue stick). The binding residues of *Pf*KRS are shown as pink sticks. The two SNPs which lie in the binding pocket, L555I and V501I, are shown as a green surface. Abbreviations: *Pv*KRS, *P. vivax* lysyl-tRNA synthetase

The 3D structures of cytoplasmic *Pf*KRS in complex with the inhibitors cladosporin (and its analogues) and a lymphoma kinase inhibitor and substrate L-Lys are available [11–14]. The tested inhibitors, particularly the cladosporin analogues, are selective towards the parasite KRS compared to its human counterpart [11–14]. *Pf*KRS is a homodimer with an aminoacylation domain and an anticodon domain. Cladosporin occupies the substrate ATP-binding site by mimicking adenosine binding while substrate L-Lys is bound to the amino acid binding site [11–14, 30]. Our analysis revealed 64 unique SNPs in *Pf*KRS (in 583 residues), with an overall low mutation frequency of 11% (Figs. 2, 3; Additional file 3: Table S3). An individual SNP in *Pf*KRS occurs in a low number of samples, i.e. at least in one sample and in a maximum of five samples out of a total of 9751. A sole conservative mutation, V78M, occurs in five samples. Individual samples also contain only one SNP, with the exception of two samples from Gambia and Ethiopia which have two SNPs each (K59M and N176S, and N286K/T and L280I respectively), indicating an overall low genetic variation. SNPs in *Pf*KRS constitute different types of residues mutating to either a hydrophobic or polar residue (Fig. 4b). Interestingly, several polar-to-charged mutations are present (S3R, T46R, N286K, N355D, N422D, N441H) and also two mutations in which charge switched from either basic to acidic or vice versa (K209E, E423K) (Fig. 4b; Additional file 3: Table S3). Structural mapping of SNPs on *Pf*KRS shows 41% and 42% mutations in the N-terminal and aminoacylation domain, respectively, with the remaining mutations in the anticodon domain (PDB ID: 4PG3) (Fig. 5a, b). Sequence alignment of *Pf*KRS and *Pv*KRS revealed 34 corresponding mutations in *Pv*KRS, and the *P. vivax* project identified six SNPs in *Pv*KRS, of which three are unique from the former 34. Interestingly, disordered residues 1–77 have 20 mutations, and so V78M, occurring in five samples, can not be adjudged to be a core or surface mutation. The overall mutation frequency is lowest for the aminoacylation domain (only 8% in *Pf*KRS and 5% in *Pv*KRS), emphasising that the catalytic domain is structurally very conserved (Fig. 5c). Overall, ~45% of mutations are conservative, and 50% lie in flexible loops. The mutations in *Pf*KRS are predominately basic in terms of charge or polar and, notably, the polar residue that mutates is mostly an asparagine (Fig. 4b).

Residues 287–354 of the aminoacylation domain (Fig. 5d; “Patch 1”) constitute the longest sequence stretch on *Pf*KRS with no mutations, and interestingly covers half of all cladosporin and L-Lys interacting residues. This stretch surrounds the inhibitor-binding pocket and also contains the majority of homodimer interface residues. Only one buried mutation, M277T/I, is present in the dimer interface that makes an H-bond with E341 of the other monomer. Mutation clusters (defined as > 3 mutations that lie in close proximity of each other on the enzyme surface) do not exist in *Pf*KRS. All cladosporin and L-Lys interacting residues are conserved. Curiously, two mutations, V501I and L555I, are present at the base of the inhibitor-binding pocket (Fig. 5e). It should be noted that these mutations are conservative hydrophobic and that they are each seen in only one sample (in samples from Mauritania and Tanzania respectively). Thus, these two mutations have very low frequency. One distant SNP, N286K/T, within 5 Å of the binding pocket appears to have no effect on the pocket’s peripheral surface. N286K is one of the mutations coexisting in the only two samples which have two SNPs. Further, residues F342, R559, K499 and F563, which have been shown in a previous study [12] to be involved in conformational changes, are conserved. Residues V328 and S344, shown in a previous study to enable drug entry into the binding pocket [11], are also conserved. Finally, the three conserved class II motifs of *Plasmodium* KRS with Human KRS [11], 276-PMMLI-281, 329-FRNE-332 and 557-ID-559 are conserved, with the exception of one mutation, M277I/T, in the first motif.

### SNP profiles of cytoplasmic *P. falciparum *and* P. vivax* prolyl-tRNA synthetase

**Fig. 6:**
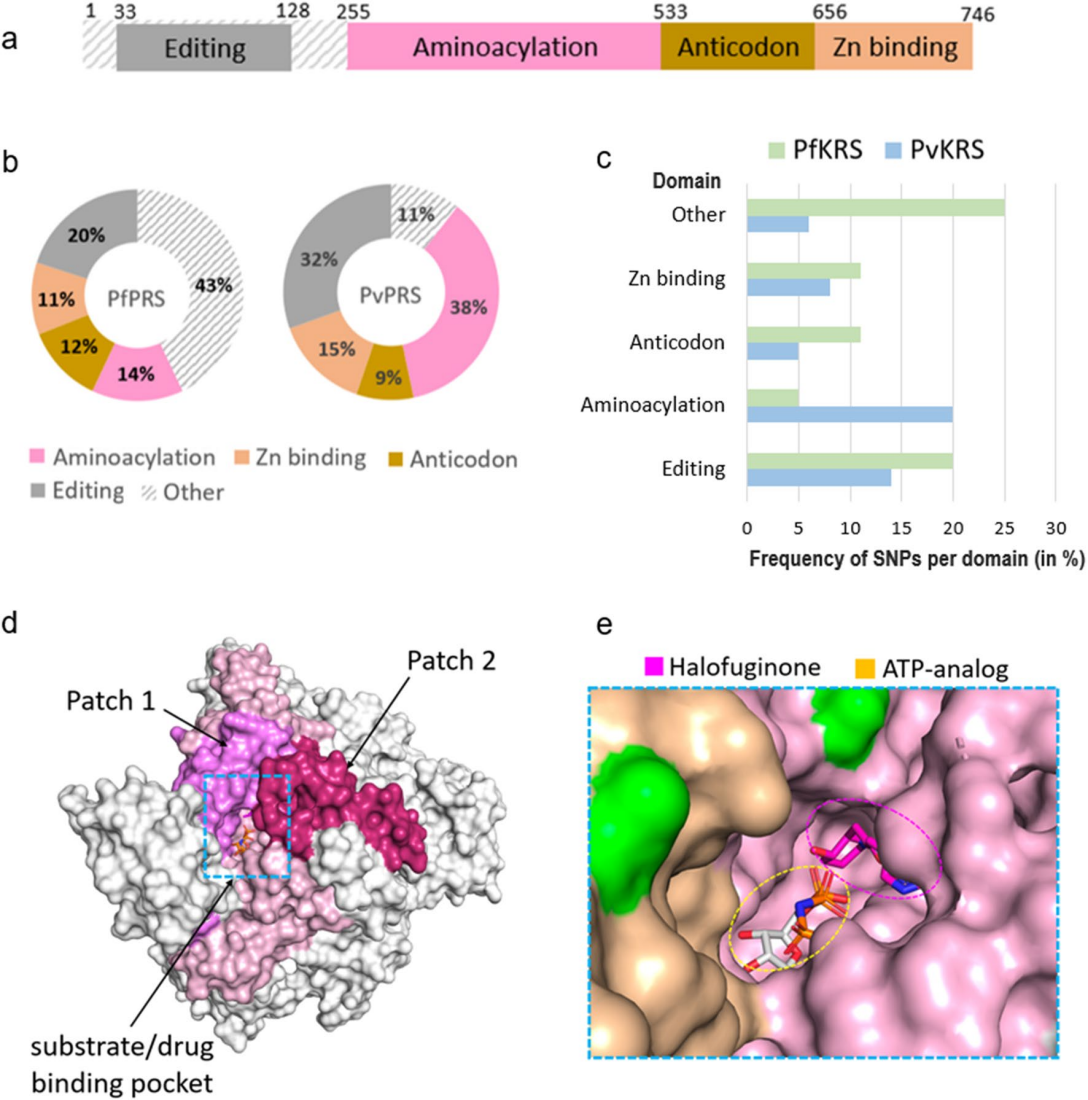
SNP profiles of cytoplasmic *Pf*PRS. **a** Domain diagram of *Plasmodium* PRS. Aminoacylation, anticodon and zinc-binding domains are shown in pink, brown and peach respectively; the editing domain is shown in grey; and the disordered region in three-dimensional structures of *Pf*PRS is shown as grey patterning. **b** The percentage distribution of amino acid mutations across the domains of *Pf*PRS (left) and *Pv*PRS (right). **c** The percentage frequency of amino acid mutations per domain in *Pf*PRS and *Pv*PRS [(total number of amino acid mutations divided by total number of residues in the enzyme) × 100]. **d** Structure of the *Pf*PRS dimer surface (PDB ID: 4YDQ). For simplicity, the aminoacylation domain of only one monomer is coloured light pink. The two sequence patches of residues having no mutations (amino acids 291–368 [Patch 1] and amino acids 418–490 [Patch 2]) are shown in magenta and violet pink respectively. The substrate/drug binding pocket is indicated with a blue box. **e** Close-up view of the primary binding pocket of *Pf*PRS bound with inhibitor halofuginone (magenta stick) and an ATP-analogue (orange stick). The proline and tRNA-binding pocket, which is occupied by halofuginone, is indicated in magenta. The ATP-binding pocket occupied by ATP-analog is indicated in yellow. The SNPs (green) are located > 8 Å from the primary binding pocket. Abbreviations:* Pv*PRS* P. vivax* prolyl-tRNA synthetase

The 3D structure of cytoplasmic *Pf*PRS in complex with inhibitor halofuginone is available (PDB ID: 4YDQ) [15–17]. *Pf*PRS is a dimer consisting of aminoacylation, editing, anticodon and Zn-binding domains (Fig. 6a). *Pf*PRS binds to halofuginone, a well-established dual-site inhibitor that binds to two sites, the 3’-end of the tRNA site and the proline site, while an ATP-analogue binds to the ATP site [15–17]. Several halofuginone analogues have been shown to have improved selectivity towards the parasite and higher therapeutic indices [17]. Also, halofuginone can be utilised in synergistic drug combination approaches, as has been shown against PRS in the parasite *Toxoplasma gondii* [31]. Our analysis revealed 93 unique SNPs in *Pf*PRS (in 746 residues), with an overall low mutation frequency of 12%. (Figs. 2, 3; Additional file 3: Table S3). Another 47 corresponding mutations in *Pv*PRS and an additional seven from the *P. vivax* project have been identified, of which five of the latter are unique from the former 47 (Figs. 2, 3). SNPs occur in at least one sample and in a maximum of 2080 samples (out of a total of 9751) in *Pf*PRS, with three mutations in particular exhibiting high occurrence: F72I (51 samples) V186I (174 samples) and V36I (2080 samples). These three mutations are conservative hydrophobic mutations and are located in the disordered region (amino acids 1–250) of the N-terminal/editing domain. Most samples have only one SNP, and only 173 samples have two coexisting mutations, which are either F72I and V186I and/or V36I. SNPs in *Pf*PRS are predominately hydrophobic-to-hydrophobic, hydrophobic-to-polar and/or polar-to-hydrophobic. Interestingly, similar to *Pf*KRS, polar-to-charged mutations involving asparagine/glutamine (N109D, Q111H, N138K, Q140R, N261K, Q395K, N416D, Q700R) are present. Also, two mutations of a switch between basic and acidic charge are seen (K92E, D561H) (Fig. 4c). *Pf*PRS has a higher number of non-conservative mutations compared to *Pf*KRS (Fig. 4). Structural mapping of SNPs on *Pf*PRS shows that 14%, 12% and 11% mutations in the aminoacylation, anticodon and Zn-binding domains respectively (Fig. 6a, b). Curiously, *Pv*PRS has a higher number of mutations in the catalytic domain compared to *Pf*PRS. The domain-wise distribution shows 13 SNPs in aminoacylation domain of *Pf*PRS, i.e. only a 5% frequency, indicating a highly conserved catalytic domain (Fig. 6c). In comparison, 20% of SNPs are seen in the *Pv*PRS aminoacylation domain.

*Pf*PRS contains two sequence stretches in its aminoacylation domain with no mutations (Fig. 6d). The first stretch (amino acids 291–368, “Patch 1”) (Fig. 6d) has only one conservative mutation, E312D, and it also comprises residues 316–351, which are involved in the ‘collapsed conformation’ that imparts a characteristic asymmetric homodimer interface [15] to *Pf*PRS (Fig. 6d). A second stretch (amino acids 418–490, “Patch 2”) (Fig. 6d) contains only the SNP G455A. Intriguingly, both these mutation-free stretches are located in the vicinity of the drug/substrate-binding pocket (Fig. 6d). With the exception of one cluster of four mutations (P369S, Y375H, R376S, N498T) that is located towards the end of the aminoacylation domain connecting to the anticodon domain, SNPs on the *Pf*PRS surface are mostly singular. Analysis showed high conservation in halofuginone and ATP-analogue interacting residues (Fig. 6e). Additionally, two crucial zinc chelating cysteine residues of human PRS in the C-terminal Zn-binding domain which are replaced by serine (S732) and threonine (T686) in *Plasmodium* [17] are also conserved.

### SNP profiles of cytoplasmic *P. falciparum *and* P. vivax* phenylalanyl-tRNA synthetase

**Fig. 7:**
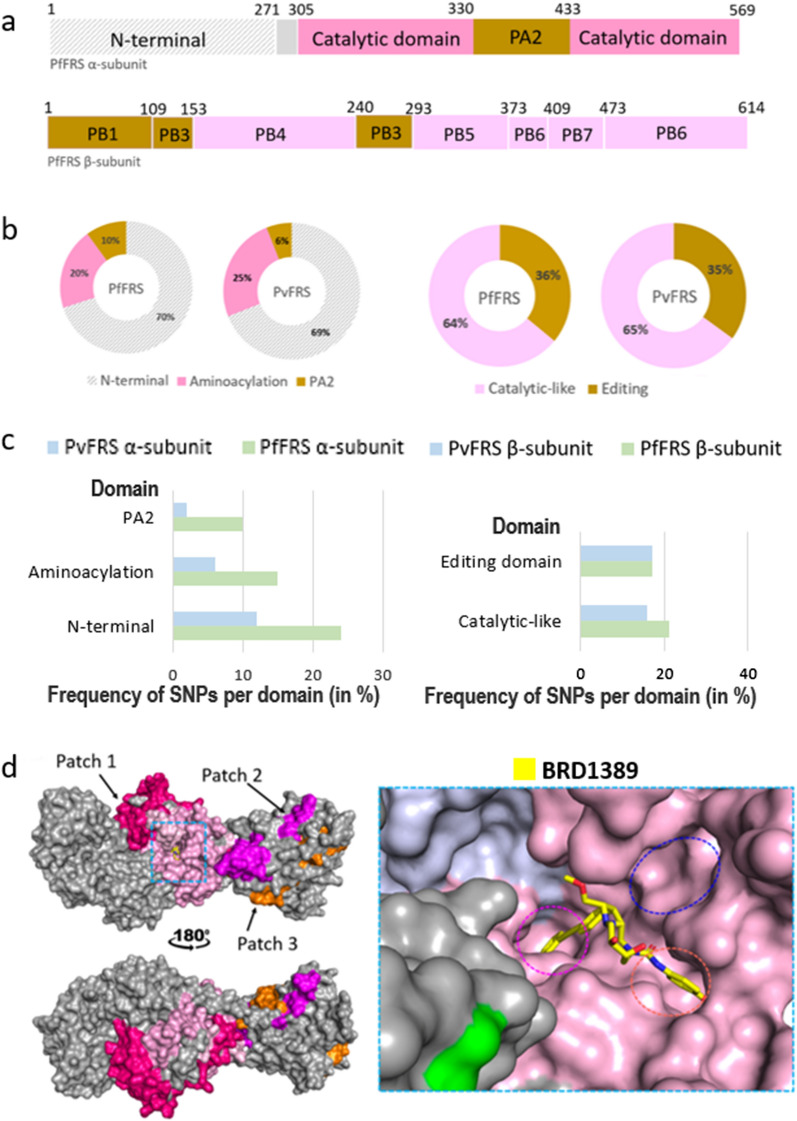
SNP profiles of cytoplasmic *Pf*FRS and *Pv*FRS. **a** Domain diagram of α-subunit of *P. falciparum* FRS. Catalytic (aminoacylation) and PA2 domains are shown in pink and brown respectively; the N-terminal is colored grey; and the disordered region in three-dimensional structures of *Pv*FRS is shown as grey patterning. Domain diagram of β-subunit of *P. falciparum* FRS. Catalytic-like and editing domains are colored light pink and brown. **b** The percentage distribution of amino acid mutations across the domains of α- and β-subunits of *Pf*FRS (left) and *Pv*FRS (right). **c** The percentage frequency of amino acid mutations per domain in α- and β-subunits of *Pf*FRS and *Pv*FRS [(total number of amino acid mutations divided by total number of residues in the enzyme) × 100]. **d** Structure of *Pv*FRS heterodimer (PDB ID: 7BY6). The α- and β-subunits are shown in grey; the aminoacylation domain of the α-subunit is colored pink; the three sequence patches of residues having no mutations [α-subunit (amino acids F313-K401; Patch 1) and two in the β-subunit (amino acids D495-R549 [Patch 2] and amino acids E567-M617 [Patch 3]) are shown in dark pink, magenta and orange respectively. The substrate/inhibitor binding pocket in the α-subunit is indicated with a blue box. **e** Close-up view of the binding pocket of *Pv*FRS bound with inhibitor BRD1389 (shown as yellow stick). The L-Phe binding pocket and an auxiliary site are indicated in magenta and orange respectively. The ATP binding pocket is indicated in blue. Abbreviations:* Pv*FRS*, P. vivax* phenylalanyl-tRNA synthetase

Phenylalanyl-tRNA synthetase (FRS) in *Plasmodium* exists as a heterodimer of α- and β-subunits that differ significantly in their sequence [18]. The α-subunit of *Plasmodium* FRS contains the aminoacylation domain while the β-subunit contains the editing domain (Fig. 7a) [18]. In the complex of *Pv*FRS and selective inhibitor bicyclic azetidine BRD1389, the drug binding region lies exclusively in the α-subunit, with BRD1389 occupying the substrate L-Phe binding site and an auxiliary site [18]. Our analysis of the *Pf*FRS α-subunit revealed 103 unique SNPs for its 575 residues, with an overall frequency of 18% (Additional file 3: Table S3). Structural mapping of SNPs on the *Pv*FRS α-subunit showed that the maximum number of mutations are located in the N-terminal region (amino acids 1–271), which is disordered in the current structure, and that ~25% of mutations are in the aminoacylation domain (PDB ID: 7BY6) (Fig. 7b, c). A particular SNP occurs in at least one sample and a maximum 266 samples (of a total of 9751 samples), and three mutations, in particular, are seen in a high number of samples: T377I (266 samples), E50K (23 samples) and N11S (23 samples). Further, every sample has only one SNP, with the exception of three samples from Mali, Ghana and Gambia, which have two SNPs each: E28V and I469V; N53K and S81T; N151S and I469V. SNPs in the *Pf*FRS α-subunit are very diverse, and the frequencies of hydrophobic-to-hydrophobic, hydrophobic-to-polar, polar-to-hydrophobic and charged to hydrophobic/polar mutations are similar. Remarkably, unlike *Pf*KRS and *Pf*PRS, a higher frequency of polar-to-charged, hydrophobic-to-charged, and switch of charge mutations are seen in the *Pf*FRS α-subunit (Fig. 4). The *Pf*FRS α-subunit N-terminal has the highest mutation frequency, and a frequency of 20% is seen in the aminoacylation domain (Fig. 7c). Up to ~50% of mutations in the aminoacylation domain of the α-subunit are conservative, and the majority are located in loops. Further, an equivalent 51 mutations are present in the *Pv*FRS α-subunit, and an additional five mutations are identified from the *P. vivax* project that are located in the disordered N-terminal. The *Pf*FRS β-subunit, which does not have a catalytic domain, has 135 unique SNPs, with an overall frequency of 22% for 617 residues (Fig. 7b, c). In the *Pf*FRS β-subunit, an individual SNP occurs in the range of one to 99 samples, with A35T and K617Q present at the highest frequency. The *Pv*FRS β-subunit has an equivalent 73 mutations and an additional 15 unique mutations from the *P. vivax* project. The maximum number of β-subunit mutations lie in β-sheets, unlike the α-subunit mutations which are typically in loops.

Three sequence stretches with no mutations are present on *Pv*FRS, with the longest stretch in the α-subunit (amino acids 313–401) (Fig. 7d, “Patch 1”) and two in the β-subunit (amino acids 495–549 and amino acids 567–617) (Fig. 7d, “Patch 2” and “Patch 3”). The α-subunit stretch surrounds the inhibitor-binding pocket from one side and is also a significant contributor to the dimer interface between the α- and β-subunits. No mutation was seen in any of the dimer interface residues. The disordered α-subunit N-terminal (amino acids 1–271) could help decipher the complete heterodimeric interface of *Pv*FRS. Also, all mutations are dispersed separately, with no prominent clustering seen on the surface. Further, the inhibitor BRD1389 interacting residues of *Pv*FRS show very high conservation (Fig. 7e). A loop (amino acids 443–453) that lies in the ATP binding pocket and ‘opens’ to enable BRD1389 [18] binding is seen to be conserved. A second loop (amino acids 507–515) that lies in the auxiliary pocket and ‘closes’ to enable binding has one mutation (A453S; hydrophobic to polar) and that is seen in only one sample (from Malawi). This A-to-S mutation retains the small side chain thereby avoiding any possible obstruction to ligand entry. Finally, four residues M310, L544, V339, and G506 near the binding pocket known to confer resistance to bicyclic azetidines [18], are also conserved.

### Drug-binding regions of validated aaRSs are conserved compared to *P. falciparum* DHPS

**Fig. 8:**
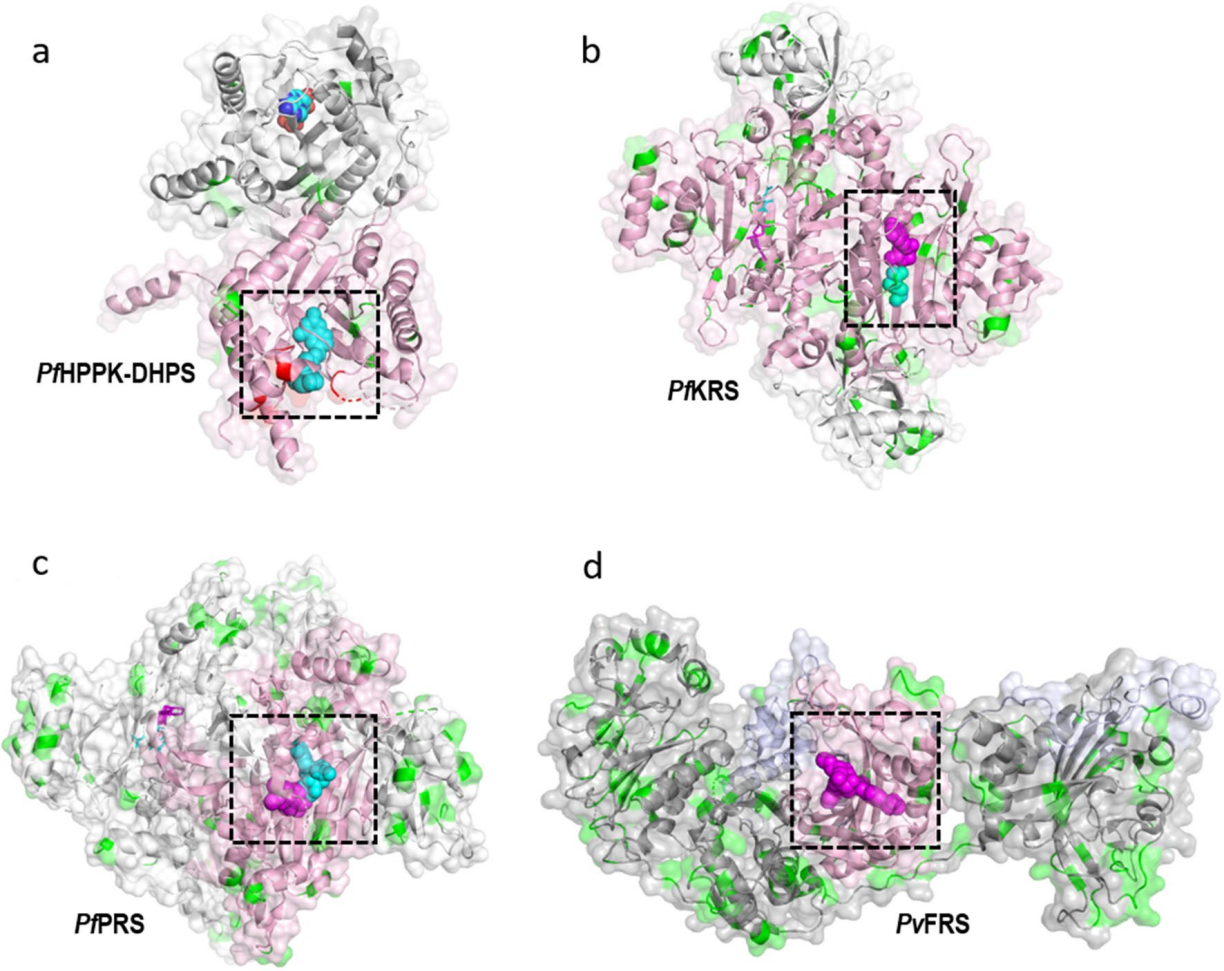
Structural mapping of MalariaGEN SNPs on four malaria drug targets. The enzymes are shown as a ribbon with a transparent surface. SNPs are colored green. Drug/substrate/inhibitor binding sites are indicated with a black box. The drug-resistance causing mutations of *Pf*DHPS domain are colored red. Drugs/substrates/ligands are shown as magenta/cyan spheres for only one monomer for simplicity. **a** *Pf*HPPK*-*DHPS, **b** *Pf*KRS, **c** *Pf*PRS, **d** *Pv*FRS

Drug resistance causing mutations in the DHPS domain of *Plasmodium* HPPK-DHPS are well-established [26, 28, 29], and our SNP analysis further confirms the existence of these mutations in the *Pf*DHPS domain in a majority of the analysed samples; however, the overall mutation frequency for the *Pf*DHPS domain was found to be only ~4% (Fig. 8a). In contrast, the drug binding regions in aaRSs show very high conservation. In *Pf*KRS, the inhibitor cladosporin (covering the ATP-binding site) and substrate L-Lys binding regions show high conservation except for two conservative mutations, V500I and L555I — both are present at the cladosporin-binding site but occur in a minuscule number of samples (Fig. 8b). The binding regions of the inhibitor halofuginone (covering the 3’-prime end of the tRNA site and amino acid proline site) of *Pf*PRS are highly conserved, with the closest mutation being at a distance of ~8 Å (Fig. 8c). Similarly, the binding region of the inhibitor bicyclic azetidine BRD1389 (covering the amino acid phenylalanine site and an auxiliary site) on *Pv*FRS is highly conserved, with the closest mutation being at a distance of  ~12 Å (Fig. 8d). Sequence and structural evaluation of SNPs of drug target aaRSs and their comparison with the DHPS domain (for which mutations causing resistance are already known) is crucial to substantiate prospects of aaRSs as successful drug targets.

Field isolate data for the *Pf*DHPS domain are available from when resistance mutations were already detected in majority of samples; it is difficult to assess whether other non-active site mutations already existed in this domain previous to the introduction of the drug [29, 32]. Resistance against antimalarial drugs remains a major concern. Resistance to inhibitors of parasite aaRSs may eventually develop; however, studies on parasite FRS indicate a low tendency to generate resistance mutations in malaria parasites [33]. The anticipated drug resistance can be delayed or partially prevented by using the drug combination approaches wherein aaRSs inhibitors can be a promising part of drug cocktails.

## Conclusions

Assessment of genetic variations caused by SNPs in validated antimalarial drug targets is vital for understanding the possibilities of drug resistance and substantiating their validation to achieve improved drug design. Aminoacyl-tRNA synthetase (aaRS) enzymes, which are essential for protein synthesis in malaria parasites, are validated and potent antimalarial targets. Our SNP analysis of *P. falciparum* and *P. vivax* aaRSs from MalariaGEN involving 9751 genome sequences from over 50 countries reveals 3182 unique SNPs in the 20 cytoplasmic *P. falciparum* aaRSs, with a mutation frequency of 9–31% for different aaRSs. In the present study, structural mapping of SNPs on the three most advanced inhibitor/substrate-bound cytoplasmic aaRSs, namely *Pf*KRS,* Pf*PRS, and *Pv*FRS, showed very high conservation in drug/substrate binding regions, with an overall low mutation frequency in the crucial aminoacylation domain and low SNP occurrence in individual samples. In comparison, the *Pf*DHPS domain of *Pf*HPPK-DHPS, another key parasitic enzyme for which drug resistance is well-established, prominently exhibits mutations that cause drug resistance despite having a low overall SNP frequency. The present study emphasises the significance of screening antimalarial drug targets such as aminoacyl-tRNA synthetases against SNPs from global databases to evaluate global genetic polymorphisms. Understanding these variances is key to strengthening drug design against validated targets and to counter resistance towards antimalarial drugs.

## Supplementary Information

**Additional file 1: Table S1.** Details of the 20 cytoplasmic and four dual-location *Plasmodium *aminoacyl-tRNA synthetases and *Plasmodium *HPPK-DHPS.

**Additional file 2: Table S2.** List of countries from where field isolate samples were collected at multiple sites for MalariaGEN projects Pf3k, Pf4.0, Pf6.0 and *Plasmodium vivax* 2016 release.

**Additional file 3: Table S3. **SNPs and the corresponding amino acid changes from MalariaGEN projects (Pf3k, Pf4.0, Pf6.0 and *P. vivax* 2016 release) in cytoplasmic *Plasmodium falciparum* and *P. vivax* lysyl-, prolyl- and phenylalanyl-tRNA synthetases and *P. falciparum* and *P. vivax* HPPK-DHPS.

## Data Availability

The dataset(s) supporting the conclusions of this article are already publicly available at MalariaGEN (www.malariagen.net). (i) [The Pf3k Project (2016): pilot data release 5 in https://www.malariagen.net/apps/pf3k/release_3/index.html] (ii) [*Plasmodium falciparum* Community Project—Catalogue of Genetic Variation v6.0 in ftp://ngs.sanger.ac.uk/production/malaria/pfcommunityproject/Pf6/Pf_6_vcf/] and Catalogue of Genetic Variation v4.0 in https://www.malariagen.net/apps/pf/4.0/]. (iii) [*P. vivax* Genome Variation May 2016 data release in https://www.malariagen.net/apps/pvgv/index.html]. The analysis material as Excel files from this study are available from the corresponding author upon reasonable request.

## Abbreviations

aaRS: Aminoacyl-tRNA synthetase
ACT: Artemisinin-based combination therapy
DHPS: Dihydropteroate synthase
MalariaGEN: Malaria Genomic Epidemiology Network
SNP: Single nucleotide polymorphism
Sdx: Sulfadoxine
tRNA: Transfer RNA
3D: Three-dimensional
