# Supplementary material for: Genomic analysis of single nucleotide polymorphisms in malaria parasite drug targets
Source: Parasit Vectors. 2022 Aug 30;15:309. doi: 10.1186/s13071-022-05422-4 (PMC9425944; doi:10.1186/s13071-022-05422-4)
Supplement: Supplementary file 1 — Additional file 1: Table S1. Details of the 20 cytoplasmic and four dual-location Plasmodium aminoacyl-tRNA synthetases and Plasmodium HPPK-DHPS. [file 13071_2022_5422_MOESM1_ESM.docx]

**Supplementary Table 1:** The details of the 20 cytoplasmic and 4 dual-location *Plasmodium* aminoacyl-tRNA synthetases and *Plasmodium* HPPK-DHPS. *The first letter of the aminoacyl-tRNA synthetase corresponds to the single letter code of the amino acid. Example, DRS is aspartyl-tRNA synthetase

| **S. No** |  | **Gene ID** | **Chromosome** | **Location** | **Localization** | **Total Non-synonymous SNPs that cause amino acid substitutions** |
| --- | --- | --- | --- | --- | --- | --- |
| ***P. falciparum* aminoacyl-tRNA synthetase (3D7 strain)** | | | | | |  |
| 1 | DRS* | PF3D7_0102900 | 1 | 128,569..131,554 | Cytoplasm | 110 |
| 2 | ERS | PF3D7_1349200 | 13 | 1,971,557..1,974,585 | Cytoplasm | 150 |
| 3 | HRS | PF3D7_1445100 | 14 | 1851656..1855054 | Cytoplasm | 307 |
| 4 | IRS | PF3D7_1332900 | 13 | 1,349,542..1,354,540 | Cytoplasm | 178 |
| 5 | KRS | PF3D7_1350100 | 13 | 2,005,394..2,008,444 | Cytoplasm | 64 |
| 6 | LRS | PF3D7_0622800 | 6 | 918,684..924,649 | Cytoplasm | 230 |
| 7 | MRS | PF3D7_1034900 | 10 | 1,383,164..1,386,554 | Cytoplasm | 157 |
| 8 | NRS | PF3D7_0211800 | 2 | 475,109..477,618 | Cytoplasm | 73 |
| 9 | PRS | PF3D7_1213800 | 12 | 589,729..593,777 | Cytoplasm | 93 |
| 10 | QRS | PF3D7_1331700 | 13 | 1,318,841..1,323,158 | Cytoplasm | 130 |
| 11 | RRS | PF3D7_1218600 | 12 | 731,430..735,541 | Cytoplasm | 53 |
| 12 | SRS | PF3D7_0717700 | 7 | 764,201..766,451 | Cytoplasm | 79 |
| 13 | VRS | PF3D7_1461900 | 14 | 2,514,219..2,518,607 | Cytoplasm | 283 |
| 14 | WRS | PF3D7_1336900 | 13 | 1,487,930..1,490,567 | Cytoplasm | 76 |
| 15 | YRS | PF3D7_0807900 | 8 | 401,761..404,190 | Cytoplasm | 32 |
| 16 | FRS alpha subunit | PF3D7_0109800 | 1 | 380,254..382,568 | Cytoplasm | 239 |
|  | FRS beta subunit | PF3D7_1104000 | 11 | 175,085..178,257 |  |  |
| 17 | ARS | PF3D7_1367700 | 13 | 2,695,379..2,700,285 | Apicoplast + Cytoplasm | 441 |
| 18 | CRS | PF3D7_1015200.1 | 10 | 614,363..618,032 | Apicoplast + Cytoplasm | 111 |
| 19 | GRS | PF3D7_1420400 | 14 | 846,155..849,672 | Apicoplast + Cytoplasm | 173 |
| 20 | TRS | PF3D7_1126000 | 11 | 1,013,901..1,017,911 | Apicoplast + Cytoplasm | 203 |
| ***P. vivax* aminoacyl-tRNA synthetase (Salvador 1 strain)** | | | | | |  |
| 1 | KRS | PVX_083400 | 12 | 150,042..152,234 | Cytoplasm | 6 |
| 2 | PRS | PVX_123380 | 14 | 1,404,490..1,407,528 | Cytoplasm | 5 |
| 3 | FRS alpha subunit | PVX_081300 | 2 | 238,364..240,073 | Cytoplasm  C | 20 |
|  | FRS beta subunit | PVX_090880 | 9 | 61,246..64,863 |  |  |
|  |  |  |  |  |  |  |
| **hydroxymethylpterin pyrophosphokinase- dihydropteroate synthase (HPPK-DHPS)** | | | | | |  |
| 1 | *P. falciparum* | PF3D7_0810800 | 8 | 547,896..551,057 | - | 33 |
| 2 | *P. vivax* | PVX_123230 | 14 | 1,256,701..1,259,581 | - | 6 |
