# Supplementary material for: Genomic analysis of single nucleotide polymorphisms in malaria parasite drug targets
Source: Parasit Vectors. 2022 Aug 30;15:309. doi: 10.1186/s13071-022-05422-4 (PMC9425944; doi:10.1186/s13071-022-05422-4)
Supplement: Supplementary file 2 — Additional file 2: Table S2. List of countries from where field isolate samples were collected at multiple sites for MalariaGEN projects Pf3k, Pf4.0, Pf6.0 and Plasmodium vivax 2016 release. [file 13071_2022_5422_MOESM2_ESM.docx]

**Supplementary Table 2:** List of countries from where field isolate samples were collected at multiple sites for MalariaGEN projects Pf3k, Pf4.0, Pf6.0 and *P. vivax* 2016 release.

| **S No.** | **MalariaGEN**  **project** | **Countries where samples were collected at multiple sites** |
| --- | --- | --- |
| 1 | Pf3k | 14 countries  Bangladesh, Cambodia, Congo, Gambia, Ghana. Guinea, Laos. Malawi Mali, Myanmar, Nigeria, Senegal, Thailand Vietnam |
| 2 | Pf 4.0 | 29 countries  Mauritania, Gambia, Guinea, Kenya, Thailand, Tanzania, Ghana, Cambodia, Mali, Papua New Guinea, Peru, Bangladesh, Malawi, Viet Nam, Colombia, Uganda, Myanmar, Laos, Congo DR, Nigeria, Madagascar, Cameroon, Ivory Coast, Ethiopia, Benin, Senegal. Ethiopia Indonesia |
| 3 | Pf 6.0 |  |
| 4 | *P. vivax* 2016 release | 13 countries  Brazil Cambodia China India Indonesia Laos Madagascar Malaysia Myanmar Papua New Guinea Sri Lanka Thailand Vietnam Bhutan Afghanistan Iran |
