# Supplementary material for: Genomic analysis of single nucleotide polymorphisms in malaria parasite drug targets
Source: Parasit Vectors. 2022 Aug 30;15:309. doi: 10.1186/s13071-022-05422-4 (PMC9425944; doi:10.1186/s13071-022-05422-4)
Supplement: Supplementary file 3 — Additional file 3: Table S3. SNPs and the corresponding amino acid changes from MalariaGEN projects (Pf3k, Pf4.0, Pf6.0 and P. vivax 2016 release) in cytoplasmic Plasmodium falciparum and P. vivax lysyl-, prolyl- and phenylalanyl-tRNA synthetases and P. falciparum and P. vivax HPPK-DHPS. [file 13071_2022_5422_MOESM3_ESM.docx]

**Supplementary Table 3:** SNPs and the corresponding amino acid changes from MalariaGEN projects (Pf3k, Pf4.0, Pf6.0 and P. vivax 2016 release) in cytoplasmic *Plasmodium falciparum* and *P. vivax* lysyl-, prolyl- and phenylalanyl-tRNA synthetases and *P. falciparum* and *P. vivax* HPPK-DHPS. *The first letter of the aminoacyl-tRNA synthetase corresponds to the single letter code of the amino acid. Example, KRS is lysyl-tRNA synthetase

| **S. No** |  | **Gene ID** | **Total Non-synonymous SNPs that cause amino acid substitutions** | **SNPs** |
| --- | --- | --- | --- | --- |
| ***P. falciparum* aminoacyl-tRNA synthetase** | | | | |
| 1 | KRS | PF3D7_1350100 | 64 | S3R, S5A, F10I, F21C, K30N, N31I, T32P, I36L, C38Y/F, H39Y, S42I, V45A, T46R, E49D, K59M/N/E, K61M, S67N, K68T, K72R, G76V/S, V78M, D93E, I99L, T109S, I112V, G123C, I132T, T136A, V155I, V163M/L, S168C, G175V, N176S, A178S/V, K209E, T211I, I212L, R268I, F271L, M277I/T, L280I, I281V, N286K/T, N355D, V369I, Y376C, P389S, I392V, E405D, I407L, N422D, E423K, N441H, A446S, H455Y, V501I, K515R, L520S, L535F, S537L, L555I, K567R/N, M579I, A582V |
| 2 | PRS | PF3D7_1213800 | 93 | L16V, K18M, E20Q, K24N, E28K/N, N32K, V36L/I, L40F, A41V, I44V, L48I, K67N, K69R, F72I, T81I, V82I, L84F, N86I, L87I, K92E, N105I, N109D, Q111H, K124T, F132L, E134D, E135Q, I136M, N138K, Q140E/R, E141D, K156N, E165Q, S166F/Y, A170S, 172G, T180P/S, T181K, S182T, K183R, A184T, V186G/I, K189N, E190Q, V192I, Q193R, M196I, E201V, Q203L/H, N214Y, N220D, L235I/V, S237C/V, N239K/I, V241A, E242Q, D243N, S249T, N250Y, N261K, A290S, E312D, P369S, Y375H, R376S, Q395K, N416D, E418K, G455A, K491Q, N498T, K527N, V535I, F546V, A554V/S, H556Y, D561H, I562V, V574I, A579P/E, C622R, V624I, D654E, S663N, N667S, M674I, A677T/V, A684G, K691N, Q700R, N702S, P722L/H, W731C |
| 3 | FRS alpha subunit | PF3D7_0109800 | 238 | N5K, E7K, N11S, E23V, E24, S28C, E31K, K33I/Q, N35I, N36D/S, K37E, D40G, G41D, D43G, D45Y, K48I, E50K, N52Y, N53K, N55K, E56V, Y57F, K59I, G66C/D, D68V, V69, L70F, N72Y, T76S, S81C/T, K90N, V91I, K96R, Y102N, L107R, D118N, P129S, D147N, K150I, N151S/K, L152F, K154R, T162I, Q170K, D175N, C177Y, I187T,  T191A, Q192H, D196Y, H203D, D204Y, A210S, H211N, N216K, H217D, T218A, K219N, K228E, N229K/I, C231Y, N235S, I236M/N/S, L240F, D243E, K253R, N255K, V260I, D269N, Q273K, Y279F, I292L, I309V, L312I, T318A, T369I, I371L/V, K373N/E, T377I, T388A/S, H389Y, K400I, E404D, K408R, A422V, I436V, K437R/N, A459S, V464I/A, I469V, K471R/N, A483E, A484S, H488Q, H492L, K493M, M508T, Q516K, E541K, V543A, K572R, S573G, V574I |
| 4 | FRS beta subunit | PF3D7_1104000 |  | S5T/P/L, E8A, F12L, G16E, E17D, E22K/V, I38T, D43V, A53S, C60F, R66L, K69N, N70Y, C73R, K74R, D77N, D81N, I82V, M84K/I, N94H, Y96F, V99L, G108R, V114A, K116R/N, N117D, D122N/H, S123I, L135V, S144T, V145A, Y153C, E165G, K166R, K167N, N182K, M184V, I187M/T, D188Y/V, F189I, K192R, L194I  K201N, K204N, K208N, I211M, N216S, Q218K, S221C, H230Y, K232R, N239T, V240I, A246S, I247V, R249K, A252V/T, Q253H/E, A255V, C260S, P269L, I273C/V/L,  Q274H, Y280F/C, E281G, Q283R, D287N, Q289K/E/H, L291I, Q297E, F298V, E304K, D313H/G, R316K, S319L/T, H323D, H327P, E328G/D, N330Y, C340S, D341N, M343V, N345I, F348V, K349N/E, V350I, T351I, A372S/P, G376A, P383S, I385V, K388R/N, V403A, C405F, T408A/I, M411I, C424S, H429P/Y, K430N, S431L, D433Y, D434E, P435R/S, L439H, D440E, A446G, A447P, I449V, Q450E, K452M, S457F, S475C, S493C, T496A, Y497F, Q499K, T500I, A504T, V505I, L510I, V528I, S540N, I552M, S553T/Y/F, R555P, S556F, E566D, L571W, N572D, I575L, F581L, V589I, V607M, A609V/S/T, L618F |
| ***P. vivax* aminoacyl-tRNA synthetase**  **SNPs from *P. vivax* project** | | | | |
|  | KRS | PVX_083400 | 6 | \| G16S, H55Y, M57L, A64P, A180S, A584V \| \| --- \| |
|  | PRS | PVX_123380 | 7 | \| S37G, L120M, G146S, A268V, S355R, A506S, D686E \| \| --- \| |
|  | FRS alpha subunit | PVX_081300 | 20 | \| L59I, I162M, E192G, P219T, F277L, K41Q, A161T, F209L, G217D.  V78I, Y109F, V114I, F164Y, T301I, F356V, F356C, L478M, V614A, K555N, M584L \| \| --- \| |
|  | FRS beta subunit | PVX_090880 |  |  |
| **hydroxymethylpterin pyrophosphokinase- dihydropteroate synthase (HPPK-DHPS)** | | | | |
|  | *P. falciparum* | PF3D7_0810800 | 33 | I4V, L22F, E71K, C77Y, K199R, K106I, S152I, E189Q, R243K, D250V, M252I, M257K, D278N, C279F, D280N, N288S, K246R, M309V, R350G, Q416L, I431V, S436F, G437A, I441M, P454T, I484T, K540I, D545N, V548I, A581G, I588F, A613S, D642N |
|  | *P. vivax* | PVX_123230 | 5 | E132G, S382C, A383G, K512E/M, A647V |
